# Intrafamilial neurological phenotypic variability due to either biallelic or monoallelic pathogenic variants in *CACNA1A*

**DOI:** 10.3389/fneur.2024.1458109

**Published:** 2024-10-02

**Authors:** Dilbar Mammadova, Cornelia Kraus, Thomas Leis, Bernt Popp, Christiane Zweier, Andre Reis, Regina Trollmann

**Affiliations:** ^1^Department of Pediatrics, Pediatric Neurology, Friedrich-Alexander Universität Erlangen-Nürnberg, Erlangen, Germany; ^2^Institute of Human Genetics, Friedrich-Alexander Universität Erlangen-Nürnberg, Erlangen, Germany; ^3^Center of Functional Genomics, Berlin Institute of Health at Charité, Universitätsmedizin Berlin, Berlin, Germany; ^4^Department of Human Genetics, Inselspital, University of Bern, Bern, Switzerland; ^5^Centre for Rare Disorders Erlangen, University Hospital Erlangen, Erlangen, Germany

**Keywords:** early-onset epileptic encephalopathy, apneic spells, optic nerve atrophy, burst suppression, developmental epileptic encephalopathy, generalized epilepsy

## Abstract

Pathogenic heterozygous variants in *CACNA1A* are associated with familial hemiplegic migraine, episodic ataxia type 2 and spinocerebellar ataxia type 6, and more recently, neurodevelopmental disorders. We describe a severe, early-onset phenotype including severe muscular hypotonia, early-onset epileptic seizures, apnoea, optic atrophy and dysphagia in three siblings carrying compound heterozygous frameshift variants in *CACNA1A*. Two male patients died at the age of 5 or 14 months of suspected SIDS or severe developmental epileptic encephalopathy (DEE) with refractory seizures and apnoea. A male child (index patient) developed severe early-onset DEE including seizures and ictal apnoea at the age of 4 weeks. Another male child developed generalized epilepsy and mild intellectual impairment in late infancy, and his mother and his maternal uncle were identified as carriers of a known *CACNA1A* pathogenic variant [c.2602delG heterozygous, p. (Ala868Profs*24)] with a diagnosis of episodic ataxia type 2. This maternal pathogenic variant c.2602delG was detected in the index patient and child 2. Trio-Exome sequencing identified an additional heterozygous pathogenic variant in the *CACNA1A* gene, c.5476delC, p.(His1826Thrfs*30) in the index patient and child 2, which was inherited from the asymptomatic father. In conclusion, the novel compound heterozygosity for two frameshift pathogenic variants, maternally [c.2602delG, p.(Ala868Profs*24)] and paternally [c.5476delC, p.(His1826Thrfs*3)] is associated with a severe phenotype of early-onset DEE. This observation highlights the necessity of additional analyses to clarify unusual phenotypes even if a pathogenic variant has already been identified, and expands the clinical spectrum of *CACNA1A*-related disorders.

## Introduction

Pathogenic, heterozygous variants in the *CACNA1A* gene, which encodes the alpha-1 subunit of a voltage-gated P/Q-type calcium channel, are known to be involved in a heterogeneous spectrum of autosomal-dominant, neurological disorders such as episodic ataxia type 2 (MIM# 108500), spinocerebellar ataxia type 6 (MIM#183086), familial hemiplegic migraine with or without progressive cerebellar ataxia (MIM#141500), and a developmental and epileptic encephalopathy (MIM#617106) (1, 2). The neurodevelopmental part of the spectrum is broad and includes mild forms of epilepsy such as absence epilepsy or focal epilepsy as well as severe epileptic phenotypes such as early-onset refractory focal epilepsy and developmental and epileptic encephalopathy (DEE) (3–7). Furthermore, bi-allelic pathogenic variants in the *CACNA1A* gene have been reported in several infants with epileptic encephalopathy of early-onset and refractory course, severe muscular hypotonia and progressive cerebral, cerebellar, and optic nerve atrophy (4, 5, 7). However, little is still known about genotype–phenotype correlations in *CACNA1A*-related neurodevelopmental disorders and DEE (3, 8).

Here, we report a rapidly progressive and fatal course of early-onset *CACNA1A* associated developmental and epileptic encephalopathy in three siblings, of which two were identified to harbor compound heterozygous frameshift variants in *CACNA1A* and one is considered to have the same genotype. The heterozygous variants segregated in the family with milder, variable neurological phenotypes, respectively.

## Materials and methods

Clinical data including findings from general and neurological examination, seizure types and frequency, response to antiseizure medication (ASM), family history, and results of (long-term) video EEG and cerebral MRI scans were obtained from patient records. All children were treated regularly in the interdisciplinary outpatient center of the Division of Pediatric Neurology at the University Hospital of Erlangen. Neuropsychological evaluation was performed by experienced occupational therapists and psychologists.

Exome sequencing of three siblings and their parents was performed at the local Institute of Human Genetics on an Illumina HiSeq 2,500 sequencer using the Twist Human Core Exome Enrichment Technology (Twist Bioscience Inc.) and processed as previously described (9). In total, 1,454 established NDD and 1,224 additional candidate genes from the SysNDD database (version from April 2021) were evaluated (10). Variants were analyzed regarding their occurrence and frequencies in population or variant databases, their segregation and their consequences as well as regarding their plausibility in light of the phenotype. Exome-based copy number variant (CNV) analysis was performed using the ExomeDepth Software (GATK). The parents’ informed consent was obtained.

## Results

Clinical characteristics of the four affected male siblings are summarized in Table 1. The leading clinical symptoms in the index case (II-4) and two of his siblings (II-1, II-2) were severe muscular hypotonia (floppy infant), plus refractory early-onset epileptic seizures and deep apneic spells, as well as optic atrophy.

**Table 1:** Patient characteristics.

Identifier	Age, gender	Genotype	Age at seizure onset	EEG and seizure type at onset	Development	Other neurological abnormalities	Epilepsy syndrome	ASM	Neuroimaging (cMRI)
Child II-1	Died at the age of 5 months, M	n.d.	Unknown	“SIDS,” SUDEP?	Severe developmental impairment	Floppy infant	Unknown	None	None
Child II-2	Died at the age of 14 months, M	Compound heterozygous p.(Ala868Profs*24)/ p.(His1826Thrfs*30)	5 months	Hypsarrythmia, deep apnoic spells	Severe developmental impairment	Blindness, severe muscular hypotonia, edema, optic atrophy, swallowing disorder (tube feeding)	DEE	PB, LEV, TPM, pyridoxine HCI, CZB, DEX, VGB	Cerebellar hypoplasia
Child II-3	10 years, M	Heterozygous c.5476delC p.(His1826Thrfs*30)	5 years (age at last visit 10 years)	Primary generalized epileptic discharges; generalized seizures, atypical absences	Mild intellectual impairment	Ataxia, mild moderate muscular hypotonia, ADHD, dizziness	PGE	LEV	Normal
Child II-4	Died at the age of 2.5 years, M	compound heterozygous p.(Ala868Profs*24)/ p.(His1826Thrfs*30)	4 weeks	Burst suppression, bilateral clonic seizures, deep apnoeic spells, myoclonic seizures, gaze rigidity	Severe developmental impairment	Floppy infant, respiratory insufficiency (NICU) severe muscular hypotonia, Blindness, optic atrophy	DEE	PB, pyridoxine HCl TPM; VGB, DEX, VPA, LEV	Normal
Mother I-2		Heterozygous c.2602delG p.(Ala868Profs*24)	2–3 years of age (ASM until the age of 6 years), no further seizures during late childhood and adulthood	n.d.	Normal	Chronic headache, ataxic gait, dizziness	None	None	n.d.
Uncle I-3		Heterozygous c.2602delG, p.(Ala868Profs*24)	None	n.d.	Normal	Ataxic gait	None	None	n.d.
Father I-1		Heterozygous c.5476delC p.(His1826Thrfs*30)	None	n.d.	Normal	n.d.	None	None	n.d.

ADHD, attention deficit hyperactivity disorder; CZB, Clonazepam; DEE, developmental and epileptic encephalopathy; DEX, dexamethasone; LEV, Levetiracetam; M, male; NICU, neonatal intensive care unit; PB, Phenobarbital; PGE, primary generalized epilepsy; SIDS, sudden infant death syndrome; SUDEP, sudden unexpected death in epilepsy; TPM, Topiramate; VGB, Vigabatrin; VPA, valproic acid.

### Child II-1

The male infant born as the first child of seemingly healthy young, non-consanguineous parents died at the age of 5 months with suspicion of sudden infant death of infancy (SIDS). Neonatal-onset muscular hypotonia was noted, but diagnostic work-up was not performed during early infancy nor *post mortem*.

### Child II-2

The male infant was born after an uneventful pregnancy. Muscular hypotonia was present from first week of life. The boy was admitted at the age of 5 months for further diagnostics and treatment of epileptic seizures, respiratory problems as well as dysphagia and severe muscular hypotonia. EEG showed hypsarrhythmia during awake state as well as during sleep. Brain MRI, metabolic work-up, karyotyping and Chromosomal Microarray Analysis did not indicate structural, metabolic or a specific genetic etiology. Based on clinical symptoms, PEHO (progressive encephalopathy, edema, hypsarrhythmia, optic atrophy) syndrome was suspected. Antiseizure medication (phenobarbital, benzodiazepines, levetiracetam, topiramate, Pyridoxin HCl, dexamethasone, vigabatrin) for prolonged and deep apneic spells and subtle seizures was without lasting effects. Tube feeding and transient mechanical ventilation were necessary. The patient died at the age of 14 months.

### Child II-3

The male child, born after an uneventful pregnancy, was referred at the age of 5 years for evaluation of an episodic, unsteady gait, dizziness and decreased vigilance. Clinical evaluation showed mild intellectual impairment and learning disability. Cranial MRI and metabolic screening findings were normal. EEG revealed primary generalized epileptic discharges. Treatment with levetiracetam, which was initiated for primary generalized epilepsy of most probably genetic origin, was successful and led to freedom from further seizures.

### Child II-4 (index patient)

**Figure 1:**
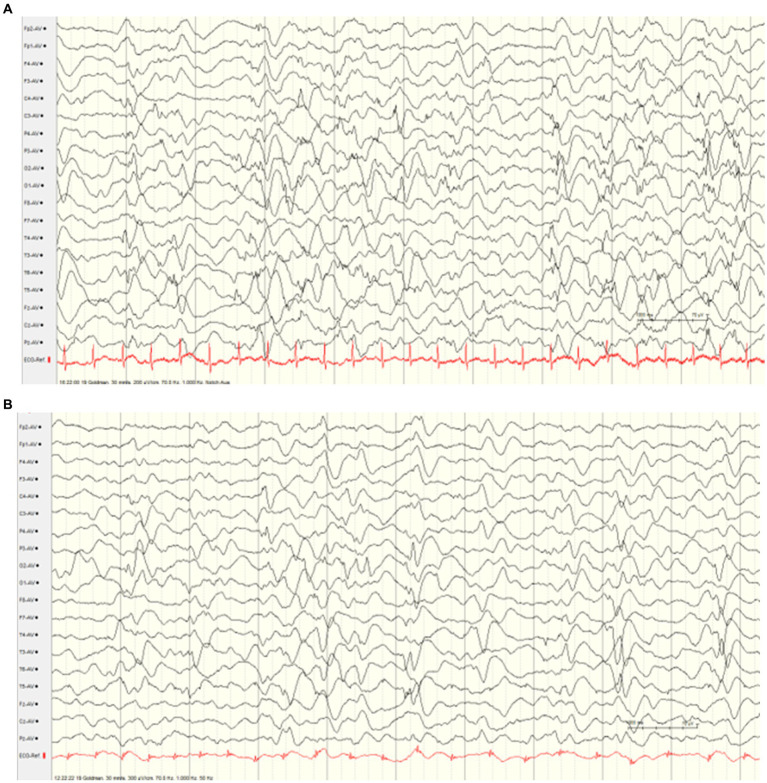
Interictal electroencephalograms (index case). **(A)** EEG at the age of 4 weeks showing burst suppression. **(B)** EEG at the age of 5 months showed generalized monomorphic background slowing and multifocal sharp waves.

The male infant was born showing a normal postnatal adaptation, however, muscular hypotonia was presented. At the age of 4 weeks, first epileptic seizures occurred, and led to the initiation of antiseizure medication with levetiracetam. However, treatment effect was transient, and the boy developed signs of an early-onset epileptic encephalopathy showing severe muscular hypotonia with absent deep tendon reflexes, respiratory and swallowing insufficiency, as well as highly frequent seizures. The semiology of the multiple daily seizures was characterized by gaze rigidity, paleness, and bilateral clonic seizures and apneic spells, as well as myoclonic seizures. Electroencephalography revealed burst suppression (Figure 1A). Cerebral MRI and metabolic work-up did not indicate any specific etiology. Antiseizure medication (including topiramate, vigabatrin, dexamethasone, valproic acid, benzodiazepines, phenobarbital) led to only transient improvement of seizure frequency with persisting severe global developmental delay and muscular hypotonia (tube feeding). EEG remained severely abnormal with generalized monomorphic background slowing and multifocal sharp waves (Figure 1B). Home care under multidisciplinary support was possible. The patient died at the age of 2 years due to respiratory insufficiency.

### Genetic results

Some years after birth of child II-3, episodic ataxia type 2 was diagnosed in the mother and one of her brothers due to a heterozygous frameshift pathogenic variant in the *CACNA1A* gene [c.2602delG, p.(Ala868Profs*24)]. The mother reported mild tremor of the hands and poor gait balance. In addition, mild intellectual deficits were apparent but not formally followed-up. The father had no specific neurological symptoms. He was heterozygous for a pathogenic variant in the *CACNA1A* gene [c.5476delC heterozygous, p.(His1826Thrfs*30)].

**Figure 2:**
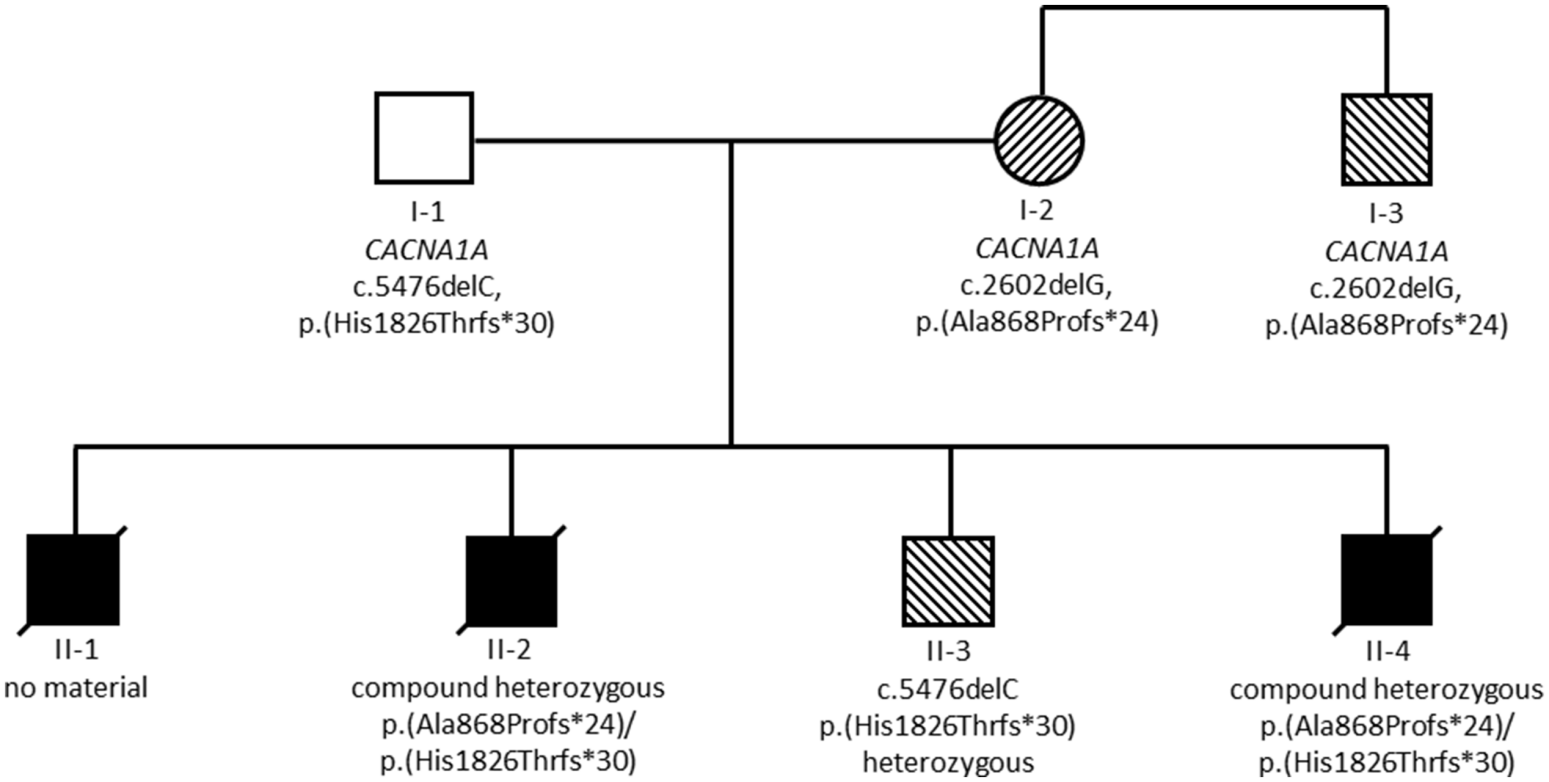
Summary of genetic results. Black symbols indicate individuals with severe and fatal developmental and epileptic encephalopathy, striped symbols indicate individuals with milder epilepsy and/or ataxia phenotypes.

The summary of genetic results is given in Figure 2. Exome sequencing revealed compound heterozygosity for two pathogenic *CACNA1A* (NM_001127222.1) variants in the index patient (II-4). Exome sequencing also confirmed these two variants in material from deceased child II-2. Child II-3 was found to be heterozygous for the maternal *CACNA1A* variant c.2602delG, p.(Ala868Profs*24). Material of child II-1 was not available for testing.

None of the two variants was included in gnomAD (11) or in ClinVar (12) databases, at this point. Both variants are predicted to result in loss of function, either by truncation or nonsense mediated mRNA decay. They were therefore classified as pathogenic according to ACMG criteria PVS1, PM2 and PP4 (13).

## Discussion

The present data indicate that the compound heterozygous frameshift pathogenic variants, namely c.2602delG, p.(Ala868Profs*24) and c.5476delC, p.(His1826Thrfs*3), cause refractory early-onset epileptic encephalopathies with a rapidly progressive course and early death. Main clinical symptoms of the severe epileptic encephalopathy in the index case [child II-4, p.(Ala868Profs*24)/p.(His1826Thrfs*30)] and in part in two of his brothers [II-1, no material; II-2, p.(Ala868Profs*24)/p.(His1826Thrfs*30)] were severe muscular hypotonia (floppy infant), refractory early-onset epileptic seizures and deep apneic spells, as well as optic atrophy. So far, only nine individuals from four families with bi-allelic variants in CACNA1 have been reported in literature (5, 7, 30, 32). Interestingly, a homozygous nonsense variant in four siblings resulted in a similarly devastating clinical course with hypotonia, epileptic encephalopathy and lethality in the first 6 months of life (32), while compound heterozygosity for a missense and a truncating variant in three other individuals, respectively, was associated with hypotonia, early-onset epilepsy, profound neurodevelopmental delay but long-time survival in two individuals (age at last assessments 10 and 5 years, respectively) (5, 7). A homozygous in-frame variant in two adult siblings was associated with progressive myoclonic epilepsy with a late onset >50 years (30) (Table 2). Our finding of bi-allelic truncating variants being associated with a severe, early onset epileptic encephalopathy with fatal outcome supports a genotype–phenotype correlation regarding deleteriousness of bi-allelic LOF variants and severity of the phenotype.

**Table 2:** Genotype–phenotype correlations in our patients and in previously reported biallelic CACNA1A variants in epilepsy patients.

References	Present Child II-2 Child II-3	(32)	(7)	(5)	(30)
Number of patients	2 (siblings)	4 (siblings)	2 (siblings)	1	2 (adult siblings)
Genotype	p.(Ala868Profs*24)/p.(His1826Thrfs*30)	c.2767C > T p.(Arg932*)	c.4315 T > A p.(Trp1439Arg) and c.472_478delGCCTTCC p. (Ala158Thrfs*6)	c.2042_2043delAG, p.(Gln681Argfs*100) and c.1693G > A, p.(Glu565Lys)	c.6975_6976insCAG
Phenotype	Early-onset developmental and epileptic encephalopathy, severe muscular hypotonia, apnoea, optic atrophy	Early-onset progressive epileptic encephalopathy, muscular hypotonia, mild facial dysmorphic features	Progressive epileptic encephalopathy, extreme muscular hypotonia, progressive cerebral, cerebellar, and optic nerve atrophy	Refractory epilepsy and developmental delay severe cerebellar atrophy by age 3 years	Refractory myoclonic seizures, neurologic deterioration and cognitive decline
Age at onset	Neonatal	Neonatal	4 months (both siblings)	6 months	56 and 54 years of age
Age at follow-up	Died at the age of 14 and 30 months of life, resp.	Died within the first 3–6 months of life	Male: 5 years Female: died at the age of 5 years	10 years	56 and 54 years of age
Mode of inheritance	Compound heterozygous	Homozygous	Compound heterozygous	Compound heterozygous	Homozygous
Functional effect	Truncating	Truncating	Truncating/missense	Truncating/missense	In-frame

Similarly to previous reports with early onset epileptic encephalopathy (5, 7, 32), heterozygous parents and one heterozygous sibling in the herewith reported family were either asymptomatic or presented with milder neurological and/or cognitive symptoms. *CACNA1* therefore belongs to the growing list of genes with co-dominantly or both autosomal dominantly and autosomal recessively inherited diseases (32).

In our patients, ictal apneic spells associated with subtle orofacial (child II-2) or epileptic spasms and generalized clonic seizures (child II-4) were the most prominent seizure type and most probably the cause of death in both brothers with the proven pathogenic variants. We also suggest that child II-1, who was diagnosed with sudden infant death syndrome (SIDS), probably died of ictal apnoea. The mechanisms of seizure-related apnoea, which is associated with a high risk of death and SUDEP (sudden unexpected death in epilepsy), including irreversible brain stem dysfunction, are not fully understood. However, animal studies suggest a lethal effect of seizures on brain stem function and respiration in adult Cacna1aS218L mice (14). These authors showed, that fatal apnea in the transgenic mice functionally characterized by gain-of-function of voltage-gated CaV2.1 Ca^2+^ channels is the consequence of cardiorespiratory dysfunction, which is induced by brainstem seizure-related spreading depolarization (SD) reaching respiratory nuclei in the ventrolateral medulla (14). Interestingly, the application of the NMDA receptor antagonists MK-801 and memantine was effective in preventing the medullary spreading depolarization and the fatal outcome (14). Data on developing transgenic mice is missing.

EEG findings were similar in the herewith reported siblings, showing a refractory suppression burst pattern from early infancy without any improvement in response to antiseizure medication. After several months, there was generalized background slowing accompanied by diffuse epileptic discharges. Similar EEG patterns were previously reported in two sisters with compound heterozygous missense and frameshift variants (c.4315 T > A), (p.Trp1439Arg); del c.472_478delGCCTTCC (p.Ala158Thrfs*6) (7), consistent with severe developmental and epileptic encephalopathy.

Pathogenicity of the identified *CACNA1* variants is strongly supported by the fact that dysfunction of the alpha-1A subunit of the Cav2.1 P/Q-type voltage-gated calcium channel causes profound functional dysregulations in various developmental neuronal pathways (15–17). The expression level of CaV2.1 α1 subunit modulates synaptic strength, efficacy of synaptic transmission and synaptic plasticity (18). While little is known about global CaV2.1 knock-out (KO) mice lacking the α1 subunit Cacna1a gene product due to early postnatal lethality, studies in a conditional Cacna1a knock-in mouse model revealed a deficiency of P/Q-type calcium channel protein and currents within the first month after birth, which is associated with altered spontaneous firing of Purkinje cells in the cerebellum and forebrain and impaired neurotransmission (17). As shown in a forebrain-specific Ca(V)2.1 KO mouse model, Ca(V)2.1 function crucially modifies complex learning and memory functions (15), and deficits are based on impaired synaptic transmission at hippocampal glutamatergic synapses.

According to gnomAD constraint scores (11), *CACNA1* is intolerant toward both missense and truncating variants (pLI = 1, z = 3.95). Accordingly, in the ClinVar database (12) both truncating and missense variants are reported across all *CACNA1A*-related phenotypes. For several missense variants, either loss-of-function or gain-of-function effects have been demonstrated, both resulting in a similar phenotype of developmental and epileptic encephalopathy (19).

Heterozygous *CACNA1A* loss-of-function pathogenic variants are rarely associated with epilepsy or epileptic encephalopathy (6, 8, 20, 21). As published by the Epi4K consortium in 2016, among 531 individuals with a variety of unresolved epileptic encephalopathy, targeted sequencing identified six infants with *CACNA1A* pathogenic variants (including an affected sibling), which were *de novo* in 3/5 families (21). Five of these infants showed first clinical seizure activity during the neonatal period including myoclonic and tonic seizure types. In addition, atypical Rett syndrome with early-onset epilepsy was associated with a *de novo* variant in the S6 transmembrane segment of domain III of the P/Q type calcium channel, CACNA1A (c.2128G > A, p.Ala710Thr) (6). Indicating intrafamilial heterogeneity, the mild cognitive impairment and childhood-onset generalized epilepsy in child II-3 was associated with a *CACNA1A* haploinsufficiency inherited from the EA2 affected mother. This is in line with previous observations of generalized absence epilepsy (8, 22–26), refractory focal epilepsy or developmental and epileptic encephalopathy (8, 21, 27) in patients with heterozygous *CACNA1A* loss-of-function pathogenic variants. Jung et al. (22) found childhood-onset refractory absence epilepsy and focal epilepsy with secondary generalized tonic–clonic seizures in 19% of individuals with proven *CACNA1A* loss-of-function pathogenic variants from four unrelated families (*N* = 3/16). A further 6 patients (*n* = 6/15, 40%) had febrile seizures during childhood. In addition to epilepsy, developmental delay, intellectual disability or executive dysfunction were found in all patients, often with ADHD (11/16) and autism spectrum disorder (4/16), as well as downbeat nystagmus and episodic ataxia in several cases. Case reports on antiseizure medication response in patients with heterozygous *CACNA1A* loss-of-function pathogenic variants show variable results ranging from seizure-free in response to pyridoxin (28), VPA and LTG (23), to partial response to VPA and LEV (22) and no response to LTG (29). The heterogeneous phenotype associated with *CACNA1A* variants is supported by experimental data on loss of presynaptic and somatodendritic CaV2.1 channel function, suggesting dysregulation of a variety of cortical and cerebellar neuronal cell types (20, 30), as well as hippocampal, thalamic and cerebellar networks (16). The present case of childhood-onset *CACNA1A*-related epilepsy with the leading symptoms of mild generalized epilepsy, mild intellectual impairment and gait ataxia highlights the variable intrafamilial phenotypic spectrum of *CACNA1A*-related diseases.

In conclusion, our observation of compound heterozygous frameshift variants in three siblings with an early-onset fatal developmental and epileptic encephalopathy further delineates the phenotype associated with bi-allelic *CACNA1* variants resulting in complete loss of function. Heterozygous presence of either variant in several family members was associated with either no or with milder, but very variable neurological phenotypes. This case highlights the need for additional analyses to clarify unusual phenotypes, even when a pathogenic variant has already been identified, and expands the clinical spectrum of *CACNA1A*-related disorders.

## Data Availability

The original contributions presented in the study are included in the article/supplementary material, further inquiries can be directed to the corresponding author.
